# Monitoring the Reaction of the Body State to Antibiotic Treatment against *Helicobacter pylori* via Infrared Spectroscopy: A Case Study

**DOI:** 10.3390/molecules26113474

**Published:** 2021-06-07

**Authors:** Kiran Sankar Maiti, Alexander Apolonski

**Affiliations:** 1 Max Planck Institute for Quantum Optics, Hans-Kopfermann-Strasse 1, 85748 Garching, Germany; kiran.maiti@mpq.mpg.de; 2 Department of Experimental Physics, Faculty of Physics, Ludwig-Maximilians-Universität München, Am Coulombwall 1, 85748 Garching, Germany; 3 Institute of Automation and Electrometry SB RAS, 630090 Novosibirsk, Russia; 4 Department of Physics, Novosibirsk State University, 630090 Novosibirsk, Russia

**Keywords:** breath, metabolites, volatile organic compound, acute gastritis, antibiotic treatment, treatment dynamics: microbiota, mid-infrared spectroscopy, short-chain fatty acid, alpha-keto acid, *Helicobacter pylori*

## Abstract

The current understanding of deviations of human microbiota caused by antibiotic treatment is poor. In an attempt to improve it, a proof-of-principle spectroscopic study of the breath of one volunteer affected by a course of antibiotics for *Helicobacter pylori* eradication was performed. Fourier transform spectroscopy enabled searching for the absorption spectral structures sensitive to the treatment in the entire mid-infrared region. Two spectral ranges were found where the corresponding structures strongly correlated with the beginning and end of the treatment. The structures were identified as methyl ester of butyric acid and ethyl ester of pyruvic acid. Both acids generated by bacteria in the gut are involved in fundamental processes of human metabolism. Being confirmed by other studies, measurement of the methyl butyrate deviation could be a promising way for monitoring acute gastritis and anti-*Helicobacter pylori* antibiotic treatment.

## 1. Introduction

A number of bacteria-related diseases increases as our understanding of the role of microbiota deepens (for detail, see Section 1 of the Supplementary Materials (SM)). One of the bacterium in stomach called *Helicobacter pylori* (*Hp*) attracts much attention [1] since its discovery in 1983 because, under some unknown circumstances it can lead to gastric problems including peptic ulcer disease [2]. It is agreed that should *Hp* be present in stomach and not eradicated in cases of related gastric problems, it can lead to gastric cancer. A common way of eradication includes a certain combination of antibiotics of broad spectra, called a quadruple course (QAC, detail in Methods). In 2011, QAC demonstrated 95% eradication success [3]. It has to be noted that the success rate of the course degrades with time because of *Hp* adaptation to the antibiotics [1,3]. The only reliable method to determine whether the bacterium is susceptible or resistant to a particular antimicrobial is to perform in vitro antimicrobial susceptibility testing [4].

Revealing the *Hp* presence via ^13^C urease or gastroscopy tests represents two established techniques for practical monitoring. Measurements with the first technique can be done either by means of mass-spectrometry or optical spectroscopy, showing similar accuracy [5,6,7]. The second technique is invasive, with clinician-dependent outcome. In both cases of *Hp*-positive tests, a clinician usually prescribes QAC. The treatment does not imply an extra step to verify the remaining *Hp* in stomach. The duration of QAC should be optimal for a given case: being too short, it does not eradicate *Hp* but makes bacteria more resistant to the antibiotics, whereas being too long, it brings negative side effects due to general harm of antibiotics on microbiota. Therefore, monitoring the progress of QAC aimed at its optimal duration would be beneficial in each individual case. In regard to the antibiotic anti-*Hp* treatment, an important question should be posed: how does QAC affect other than *Hp* bacteria? The urease test cannot answer this question because of its specificity to *Hp*. It was reported that about 30% of bacterial species were influenced by the treatment with ciprofloxacin [8]. Although most bacterial groups and subgroups (called strains; diversity within the bacterial gene) recovered after treatment, several of them did not, even after six months. Controlling their recovering is an important practical task not solved so far, to the best of our knowledge [8,9]. For cases not related to *Hp*, the recovery process was recently modeled [10]. 

There are three more powerful techniques for detecting bacteria that are not (yet) of practical use for *Hp*: serum antibody test, quantitative polymerase chain reaction (qPCR) [11] and 16S rRNA sequencing [12]. The restricted practical application of the latter technique is caused by its high price and the fact that 16S rRNA sequencing has some limitations [12] including the accuracy [13] and the necessity to use one pipeline for an accurate comparison of the data. The sequencing applied to *Hp* in feces [14] and other bio-samples [15] already revealed detailed information about the *Hp* strains and migration in the stomach for a steady state in the body. We are not aware of any study with this technique focused on the dynamics of anti-*Hp* treatment. Variations of bacterial concentrations caused by an antibiotic treatment have already been analyzed [8] in feces, resulting in highly diverse effects for three volunteers. The authors found a substantial, but not full, return to the pretreatment feces composition within 4 weeks after the treatment. 

Another, technically more practical, way of monitoring the state of microbiota could be to measure the products of its metabolism. Among them, volatile products extracted from headspace of urine, blood (including breath), or feces [16] have attracted much attention. The corresponding measuring techniques such as e-nose [17] and laser-induced breakdown spectroscopy [18] were recently tested. One study based on mass spectrometry gas chromatography was focused on analysis of breath variations caused by anti-*Hp* treatment [19]. The study revealed certain (but not significant) differences in the volatile organic compound (VOC) content prior to anti-*Hp* treatment and after. The authors concluded that their observation could be explained either by the action of antibiotics on the gut microbiome, or by the effect of the probiotics rather than the presence or absence of *Hp*. 

Bearing the current situation in mind, we suggest that measurements prior, during, and after the antibiotic treatment should be beneficial. Being combined with the steady-state data of the individual [20], such a study could unambiguously reveal the effect of the treatment.

There is growing understanding that the response of a subject to antibiotic treatment is unique [8,10]. The response is the result of at least two major factors: the gut microbiome content in general and the history of previous antibiotic treatments. The first factor defines a list of bacteria present in the gut together with their strains. It is important to note that strains of the bacterium have different sensitivity to antibiotics [21]. So far, the second factor was demonstrated experimentally only for mice. The main conclusion was that antibiotics reduce or eliminate most products of bacterial metabolism including short-chain fatty acids (SCFA) [22]. 

In comparison to analysis of feces mentioned above, breath could have several advantages: (a) it allows to make more frequent monitoring of the metabolic state of bacteria and (b) it carries direct information about the gut microbiome state. To note, bacteria in feces become already modified in comparison to the gut state and, strictly speaking, should be considered as being measured in vitro [13]. 

The aim of this one-case study was to verify whether breath carries significant information about acute gastritis and the dynamic response of the body to anti-*Hp* treatment. 

## 2. Working Hypotheses, Results, and Discussion 

The results presented below are aimed at verifying four working hypotheses related to QAC. They can be formulated as follows: (1) a conventional anti-*Hp* course based on antibiotics of broad spectrum kills *Hp* as well as other bacteria in the entire gastro-intestinal system (inspired by [8]); (2) different types of bacteria have different resistivity and reaction to antibiotics, depending in the same time on the individual. The bacterial resistivity can be so high that the treatment cannot affect them; (3) bacterial groups affected by antibiotics of QAC but present in a parallel probiotic course, should show fast recovering; (4) in order to see the treatment dynamics, monitoring must be provided not only before and after the course but also during it. The expected corresponding outcomes of these hypotheses are the following (referring to the hypothesis numbering): (1) and (2) monotonic decay of the concentration of different types of bacteria on different time scales; (3) significantly different time scales of the microbiota recovering. For volatile products related to bacteria strains present in the Omni-biotic 10 probiotic course [23], one can expect their recovering on the time scale of the course. For volatile products of the bacteria out of the Omni-biotic 10 course, one can expect slower recovering on a long time scale of months [24,25] or even years [8,10]. The hypotheses define the time scale of the study we aimed for. Specifically, a period of several months before and after the treatment should be used to collect the data regarding the steady state of the body. The absence of the steady state would mean that the hypotheses we formulated cannot be verified. Deviations of VOCs originated from bacteria sensitive to QAC, must be synchronized with the beginning and the end of the treatment.

**Figure 1:**
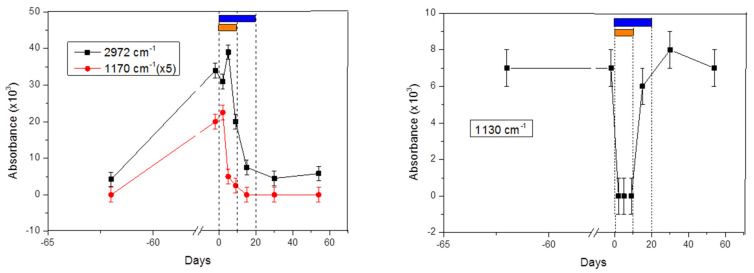
Absorbance variation (proportional to the concentration variation) of three spectral structures centered at 2972 cm^−1^, 1170 cm^−1^ (**left**), and 1130 cm^ß1^ (**right**) caused by QAC. The lines connecting the data points are used for better visibility. Vertical dash lines show the start of the QAC course (left line), the end of the antibiotic course (middle line) and the end of the probiotic course. The corresponding bars indicate the same: orange horizontal bar shows the antibiotic treatment in frame of QAC whereas blue bar—Omnibiotic 10 course taken in parallel to QAC. Absorbances at −62 and 58 days at the plots correspond to the steady state level of the corresponding VOC. Data points corresponding to dates earlier than −60 and longer than 60 days were collected and used only for analysis; they are not presented here in order to improve the visibility of the plots.

**Figure 2:**
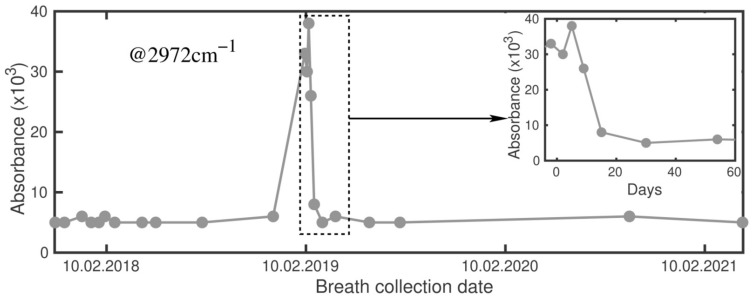
Variation of the absorbance centered at 2972 cm^−1^ during a period of 3.5 years, with the peak related to accute gastritis and QAC. Inset: the recovering dynamics via QAC.

Figure 1 demonstrates variations of the absorption signals from breath samples before, during and after QAC. To note, the signals are proportional to concentrations of the corresponding VOCs. First, we see that in both plots corresponding to different spectral ranges, the steady state (i.e., the same absorbance level before and after QAC) does exist. A correlation of both signals with the beginning and the end of the treatment allowed us to surmise that they originate from bacteria. Figure 2 represents an extended illustration of the steady state found in [20], related here to the signal at 2972 cm^−1^ (the left plot in Figure 1). Analysis of the identified metabolites corresponding to the both plots also revealed their bacterial origin (Section 3.4).

Second, in both plots of Figure 1 we see a ten-fold variation of the absorbance caused by acute gastritis and QAC, significantly exceeding natural variations of the steady state. One can also see that the characteristic time scales of the detected signals vary from few days (the right plot) to approximately 20 days (the left plot). The result correlates with the available literature data [8,9]. Different time constants identified from the two plots, call for considering two classes of bacteria affected by QAC, namely semi-resistant (the left plot) and susceptible (the right plot).

### 2.1. Signals at 2972 cm^−1^ and 1170 cm^−1^

Two signals on the left plot of Figure 1 demonstrate qualitatively similar variations from the steady state level, with well-synchronized onsets of their decays. The steady state level of the subject was defined by the data used in Figure 2. Time series data became possible in frame of another study [20] and here we used the extracted values corresponding to the period out of QAC. Significant elevation of the signal just before QAC started (day “−2”) was attributed to acute gastritis. That day, because of extra pain in stomach, the subject visited a doctor who recommended immediate QAC. Because of their slow reaction to QAC, the signals were linked to the product (-s) of semi-resistant bacteria. Their decaying part was attributed to the main eradication effect of QAC. We do not attribute the elevated point on day “−2” to *Hp* because of two reasons: based on the anamnesis, the corresponding infection occurred many decades ago. The second reason is discussed in Section 2.6. The findings shown in Figure 1 support the first two working hypotheses.

### 2.2. Signal at 1130 cm^−1^

The signal starts recovering to its steady-state level right at the end of the antibiotic part of QAC and finishes at the end of the probiotic part. It indicates that it is related to the bacteria present in the QAC probiotic part [23] described in the Methods. The abruptly decaying signal in the beginning of the treatment (day “0” to day “10”) indicates that the effect of antibiotics on the corresponding bacteria is significantly stronger than the effect of probiotics. Potential candidates for such bacteria are discussed in Section 3 of the Supplementary Materials.

### 2.3. Other Signals

We also analyzed the structures centered at 1189 and 1203 cm^−1^. They found to be insensitive to QAC. The first one was attributed to the mixture of ethyl and propyl propionates in our previous study [20]. Propionic acid is the product of bacteria responsible for food fermentation (Section 3 of the Supplementary Materials) and represents one of the main SCFA in the body. The second structure was attributed to ethyl vinyl ketone, an oxygenated hydrocarbon lipid molecule. The two types of molecules support the second working hypothesis.

### 2.4. Identification of the Molecules Responsible for the Spectral Signals

**Figure 3:**
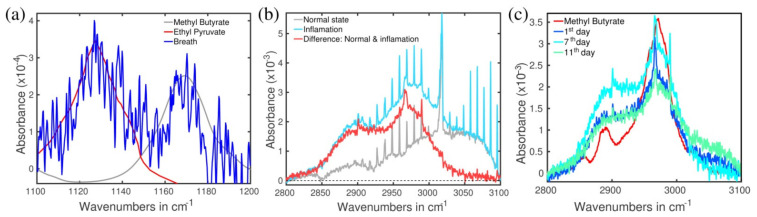
(**a**) Breath absorption spectra at 1130 cm^−1^ together with the ethyl pyruvate spectrum (red) taken from [27] as the best fitting candidate, and at 1170 cm^−1^ together with the methyl butyrate [27] (grey). The spectra correspond to day “−2” (the first elevated points on the left plot of Figure 1). Noisy signals are caused by the presence of residual water. (**b**) The difference (red) between the inflammation (day “0”, turquoise blue) and normal state (day “10 February 2018”, gray). Sharp spikes in the turquoise blue and gray curves belong to methane. (**c**) Differential (see text) breath absorption spectrum taken during the antibiotic treatment together with the measured methyl butyrate absorption spectrum as the best fitting candidate (red).

Figure 3 represents the structures of the absorption spectra that were used for the temporal analysis in Figure 1. It turned out that the three spectra correspond to two VOCs, namely methyl butyrate (1170 cm^−1^ and 2972 cm^−1^) and ethyl pyruvate (1130 cm^−1^). In plot (a), one can see that the fitting quality, in spite of weak absorbance and high noise, allowed to consider methyl butyrate and ethyl pyruvate responsible for the detected structures. Structures at 1130 cm^−1^ and 1170 cm^−1^ were revealed by digital removal of the above-lying structures that belong to carbon dioxide and aldehydes [26]. They were then identified using the two-step procedure described in Methods (without step 3). Fitting the structures with the spectra taken from the NIST database [27] led to the agreement within 3 cm^−1^ (0.3% inaccuracy). Plot (b) represents the difference between the inflammation (acute gastritis) and the healthy state. The subtraction procedure removed the structures with sharp spikes that belong to methane. We used the fact that methane concentration was constant during the entire period shown in Figure 2. Plot (c) presents the spectral structure centered around 3000 cm^−1^ that we revealed in the following way: the subtracting procedure was applied to the data taken in different days of QAC. In this step, we took the spectrum measured 30 days after the start of QAC as a reference, subtracting it from the spectra measured during the QAC period. A similar result was obtained when the spectra measured 60 and 192 days prior QAC were taken as the reference. Subtraction of one reference data from another gave a flat line supporting our suggestion that only the body reaction to QAC should be revealed via such a procedure. In general, identification of spectral structures around 3000 cm^−1^ is difficult: hundreds of VOCs have fundamental absorption bands there caused by C-H bonds. A synchronous variation of the entire structure between 2940 cm^−1^ and 3016 cm^−1^ for different days along QAC (Figure 3) supports a suggestion that in our case it mainly represents one VOC. By applying a three-step identification procedure (see the Methods section), we concluded with high probability that the structure belongs to methyl butyrate. 

Methyl butyrate—The detailed analysis of the spectral structures shown in Figure 3 provides four reasons to trust the identification: it (a) fits narrow, far-separated peaks at 1170 cm^−1^ and 2972 cm^−1^ with the accuracy of better than 6 cm^−1^ (i.e., relative inaccuracy 0.2%), (b) perfectly matches the main broad peak 2946–2992 cm^−1^, (c) perfectly fits the total spectral structure of approximately 200 cm^−1^ width between 2840 and 3040 cm^−1^, (d) demonstrates similar asymmetry of the absorption peak (the low-wavenumber tail). Nevertheless, we do not exclude contributions of other molecules to the experimental peak in the range 2850–2940 cm^−1^. Methyl butyrate was reported in the compendium for breath and feces [16], whereas according to HMBD [28], it was previously found only in feces. It is a product of bacterial metabolism in the gut (detail in Section 3 of the Supplementary Materials). 

Ethyl pyruvate—The metabolite represents a derivative of another class of acids, namely alpha-keto acids. It has been identified in [28] but is absent in the compendium [16]. The latter could relate to the fact that pyruvic acid is not an end product but rather a source for other metabolites like SCFAs, carbohydrates, etc. (detail in Section 3 of the Supplementary Materials).

### 2.5. Type of Vibrations Attributed to the Characteristic Spectral Structures of Ethyl Pyruvate Observed at 1130 cm^−1^ and Methyl Butyrate at 1170 cm^−1^

Whereas the vibration around 3000 cm^−1^ is mainly defined by the C-H bond resulting in a difficulty of the molecular identification, the vibrations of ethyl pyruvate at 1130 cm^−1^ and methyl butyrate observed at 1170 cm^−1^ turned out to be very specific to these molecules. Numerical simulation (Section 3.4 of the Methods) allowed us to identify the corresponding vibration modes. In both cases, they were identified as a combination of C-O stretching and C-H bending modes involving the movement of the entire molecular skeleton. For methyl butyrate, the vibration was identified as a combination of a very strong backbone C-O stretching mode and the C-H bending mode in the methyl group attached to the C-O bond. These two modes affect the movement of the other part of the molecule, and all the C-H bonds undergo some kind of bending motion. The backbone of the molecule also shows bending motion restricted to the plane of the backbone.

**Figure 4:**
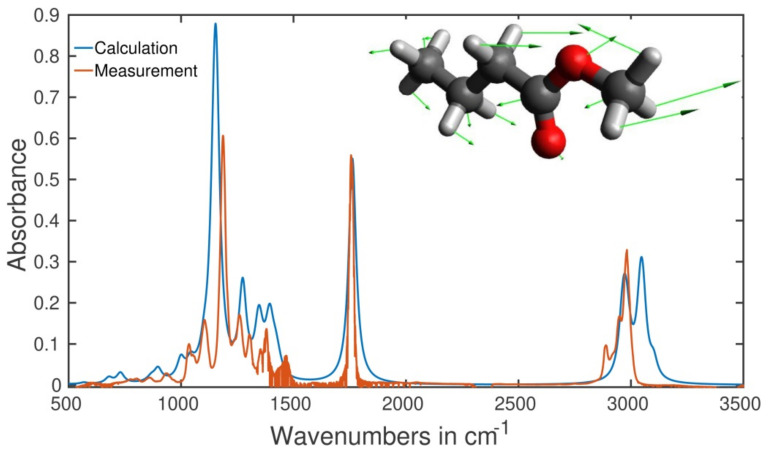
Orange curve: measured absorption spectrum of methyl butyrate; blue curve: result of numerical calculation (see Section 3.4). Inset: 3D structure of methyl butyrate. Green arrows show the movement of atoms in the complex vibration linked to the peak at 1170 cm^−1^. Red balls: oxygen, large grey balls: carbon, small grey pins: hydrogen.

For illustration, the retrieved absorption spectrum of methyl butyrate is shown in Figure 4, together with the measured spectrum. Their good qualitative agreement in the entire fingerprint region can be considered as another confirmation of validity of the identification. Shifts in positioning for the peaks centered at 1170 and 3000 cm^−1^, observable for the calculated and measured spectra, represent a general problem for any quantum chemical calculation. We consider the agreement as another evidence of the power of gas phase spectroscopy in terms of accuracy of molecular identification. The identified complex vibration, unlike low-specific single C-H, C-O, or C=O modes used in analyses of biofluids in liquid phase, characterizes the molecule in a unique way because all the molecular skeleton is involved (see the arrows in the inset). 

### 2.6. A Hypothesized Transportation Scheme and Parent Bacteria

A possible origin and transportation of ethyl pyruvate and methyl butyrate in the body until their extraction in the lung alveoli are discussed and illustrated in Section 4 of the Supplementary Materials. Their parent bacteria are discussed in Section 3 of the Supplementary Materials. Butyric and propionic acids and their derivatives, in our case methyl butyrate and ethyl/propyl propionate, are absent in the list of main metabolomic pathways of *Hp* (see figure 2 in [29]). As ethyl pyruvate demonstrates variations only during QAC but not after (the right plot of Figure 1), we also do not attribute this metabolite to the product of *Hp*. It allowed us to consider the identified metabolites as suitable for monitoring bacteria other than *Hp*, being affected by anti-*Hp* antibiotic treatment. 

## 3. Materials and Methods

### 3.1. The Instrument

We used Bruker FTIR spectrometer Vertex 70 based on thermal source, operating in the mid-infrared spectral range of our interest 500–4000 cm^−1^ (2.5–20 µm). The spectral resolution 0.5 cm^−1^ was kept for all the measurements. Gas samples were collected in single-use Tedlar bags (Sigma Aldrich) and measured then by using a system that significantly suppresses the amount of water vapor [30]. The minimum detectable concentration (the detection noise) reached 50 ppb (part-per-billion, Section 2 in SM). This value corresponds to 1 × 10^−4^ of the absorbance at 1100 cm^−1^ in Figure 3a. The absorbance units were used throughout the text because they represent physical values measured in the experiment. Their transformation into concentrations can be done only if the corresponding spectral structure is identified as a certain VOC. 

### 3.2. Subject of the Study and a Description of the Treatment Antibiotics Course 

The breath content of a 64-year old men participating in this study was monitored for more than three and a half years, including several snapshots during and after the antibiotics course. The reason for the QAC course was acute gastritis. Gastritis started in his childhood, with different periods of pain intensity. His visit to gastroenterologist revealed a distinct epithelial area affected by inflammation corresponding to the *Hp* activity. The following QAC assisted by the probiotic course was monitored by five measurements shown in Figure 1 and Figure 2. The QAC included: a 10-day course of pump inhibitor (PPI, esomeprazol), tetracycline, metronidazole, bismuth citrate accompanied by a 20 day of the probiotic yeast course (Omni-biotic 10). PPI decreases the acid production in stomach and possibly, adhesion of *Hp* to stomach epithelium. Omni-biotic 10 contains 10 human bacterial strains [23]: *Lactobacillus acidophilus W55, Lactobacillus salivarius W24, Lactobacillus acidophilus W37, Lactobacillus plantarum W62*, *Lactobacillus paracasei W72*, *Bididobacterium bifidium W23, Lactobacillus rhamnosus W71, Bifidobacterium lactic W18, Enterococcus faecium W54, Bifidobacterium longum W51.* No post-treatment healthy check was performed. 

### 3.3. Identification Procedures of the Spectral Structures

Identification of molecules was done according to the three-step procedure developed in [26,31], with the help of theoretical hints developed in [32]. The hints make a link between a 3D molecular structure and the shape of the corresponding absorption spectral structure. It has to be noted that the identification is a high probability guess, bearing in mind a large number of molecules with the absorption bands in the range of our interest, especially around 3000 cm^−1^. The range is defined by C-H vibrations common for all organic molecules. In short, the identification steps included comparison of the absorption spectra of VOCs from the compendium [16] with the experiment by using NIST database [27] (step 1). Candidates chosen in such a way were then measured with the spectrometer under identical technical parameters as breath samples (step 2). The final proof consisted in comparison of the remaining candidates with HMBD [28] (step 3). The accuracy of the identification of molecules achieved in our spectroscopic experiment approached 99.8% in terms of the peak position and characteristic width. 

### 3.4. Numerical Simulation 

Numerical simulation of the equilibrium structure of the identified gaseous metabolites has been performed based on the density functional theory with the Perdew–Burke–Ernzerho functional using Gaussian 09 computational chemistry software [33,34,35,36]. 

## 4. Conclusions and Future Work

To the best of our knowledge, we demonstrate the first signatures of breath-related dynamics of acute gastritis affected by the quadruple antibiotics course. The carriers of the dynamics were identified as volatile derivatives of SCFA and alpha-keto acid. They represent the products of bacterial metabolism in the gut. As SCFAs play an important role in the regulating energy metabolism and energy supply, maintaining the homeostasis of the intestinal environment [37,38,39], their variations found in this study reflect fundamental changes of the body state caused by acute gastritis and antibiotic treatment. A direct link between SCFA, alpha-keto acid, and acute gastritis has to be clarified in further studies.

In our opinion, at this promising stage it makes sense to continue work on (1) identifying new spectral ranges sensitive to QAC; and (2) verifying the observation reported here, with conventional techniques where possible. An example for the latter could be a biochemical analysis of feces, even it is not instant in comparison to breath. Collecting the statistical data of the reaction of patients treated with QAC, with its further analysis should be considered with a precaution: a reaction of a human body to the same medication is specific, especially for non-targeted bacteria (i.e., excluding *Hp* in our case). Diverse effects in reactions of individuals to the same antibiotic treatment observed in [8,10] may be considered as an illustration of this concern. It has to be noted that, for the targeted bacteria (*Hp* in our case), the observed diversity of human reactions is low. Instead of statistical analysis, we are in favor of implementing bio-passports introduced previously [20]. They should contain individual information about the state of microbiota together with all previous reactions (in the sense of this study) to any antibiotic treatment. In our opinion, such a strategy should replace the statistical approach in both cases of analysis and treatment by introducing a personalized approach, with higher expected outcome.

As the metabolism of each bacterium is complex depending on its strains, there is no direct link between the production of a certain SCFA or alpha-keto acid, and the bacterium. Nevertheless, if the ratio of concentrations of acetate, propionate and butyrate deviates significantly from the norm 60:20:20 in the gut [37,38], it may be considered as a sign of abnormality. In our case, the reconstructed propionate/butyrate ratio of concentrations before and after QAC was found to be equal to 1, supporting the evidence of norm [37,38].

## Data Availability

Data available on request from the authors.

## Supplementary Materials

Figure S1: Left: measured 1/noise vs square root of number of scans. Right: measured 1/noise vs spectral resolution in cm−1, Figure S2: Working hypothesis of transportation of methyl butyrate and ethyl pyruvate in the body, with their release via exhaled air in lungs. References [40,41,42,43,44,45,46,47,48,49,50,51,52,53,54,55,56,57,58,59,60] are cited in the Supplementary Materials.

## Sample Availability

Samples of the compounds are not available from the authors.
