# Supplementary material for: Monitoring the Reaction of the Body State to Antibiotic Treatment against Helicobacter pylori via Infrared Spectroscopy: A Case Study
Source: Molecules. 2021 Jun 7;26(11):3474. doi: 10.3390/molecules26113474 (PMC8201021; doi:10.3390/molecules26113474)
Supplement: Supplementary file 1 [file molecules-26-03474-s001.zip › molecules-1192125-supplementary.pdf]

# Supplementary Materials

# Monitoring the reaction of the body state to antibiotic treatment against *Helicobacter pylori* via infrared spectroscopy: A case study

**Kiran Sankar Maiti[1,2] and Alexander Apolonski[1-4]**

1. *Max Planck Institute of Quantum Optics, D-85748 Garching, Germany*
2. *Ludwig Maximilian University of Munich, Am Coulombwall 1, D-85748 Garching, Germany*
3. *Institute of Automation and Electrometry SB RAS, 630090 Novosibirsk, Russia*
4. *Novosibirsk State University, 630090 Novosibirsk, Russia*

## 1. A state of microbiota and its relation to diseases

The ecosystem of microbiota attracts lots of scientific and medical attention in the last decade [40–45]. It is agreed that microbiota has diverse functions in the body, with many examples revealed for the gastro-intestinal tract, the immune system, the brain functioning etc. [46,47]. Many diseases are correlated with the variation of a certain bacterium, with only two examples of Helicobacter pylori (Hp) in case of gastric diseases [2], and *Faecalibacterium prausnitzii, Escherichia coli, Clostridioides difficile* in case of Crohn's disease [48]. It was found that microbiota is stable on a long time scale [47,49] and unique [42] for each individual. The largest number of bacterial species was found in the gut (approximately 500) and oral cavity (200) [40], whereas in stomach only dozens including *Hp* [42]. The scenario of coexistence of different species is extremely diverse including competition and co-operation [43]. A simplified competition includes fight for nutrients and space spot (niche occupation) whereas co-operation includes for example, generation of specific VOCs by a bacterium as distance-dependent danger alarm for other bacteria [43]. Stability of the complex ecological mocrobiota system can be disturbed by several factors including disease, stress or medication. The strongest example of the latter is antibiotics, with broad spectrum of harmful side effects [50]. The corresponding examples are diverse, with direct consequences lasting months and indirect that are difficult to forecast [8,9,50,51]. The diversity of antibiotic side effects depends on such factors as the body state, type of antibiotics, the duration of the treatment etc.

## 2. Minimum concentration detectable due to noise.

Experimental dependencies of the 1/noise that defines minimum detectable concentration, on the number of scans and spectral resolution are shown in Figure S1.

For the spectrometer, a noise level of

$$noise = 2.5 \times 10^{-4} / (res \times nsc)^{1/2}$$

was measured, where res is the spectral resolution in $cm^{-1}$ and nsc is the number of scans. At a resolution of 1 $cm^{-1}$ a scan takes about 1 s. Applying a signal to noise ratio of three, a minimum detectable concentration in ppm (parts per million) is given by

$$c_{min} = 3 \times noise / [absorbance\ (ppm\ m) \times L] \qquad (2),$$

where L is the absorption path length in m. For a strongest absorption line with an absorbance of $5 \times 10^{-4}$ (ppm/m), at a resolution of 1 $cm^{-1}$ and the 4 m path length of the multipass cell, equation (4) gives a minimum concentration of 375 ppb $nsc^{-1/2}$. With 100 scans (that take about 100 s at 1 $cm^{-1}$) the minimum detectable concentration is about 50 ppb.

It has to be noted that the minimum detectable concentration depends on the spectral range. For example, the range 1100-1250 $cm^{-1}$ is "noisy" because of the water and $CO_2$ absorption bands. This leads to lower spectrometer sensitivity or, alternatively, to higher minimum detectable concentration.

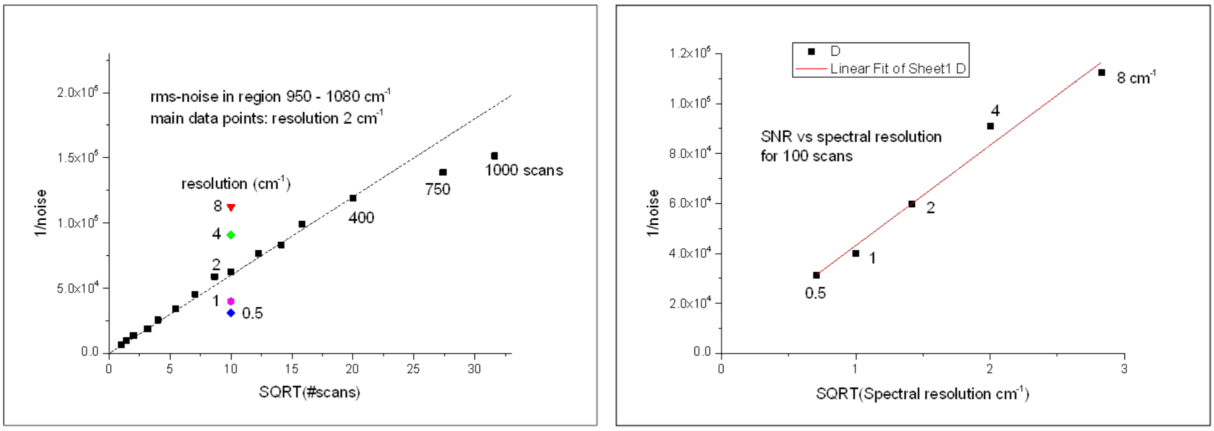


Figure S1. Left: measured 1/noise vs square root of number of scans. Right: measured 1/noise vs spectral resolution in $cm^{-1}$.

**3. Origin of main short-chain fatty acids and their derivatives in the body.**

<u>3.1. Pyruvic acid</u> (PA) is a short-chain fatty acid produced by anaerobic bacteria in the gut via fermentation of glucose through glycolysis. It is known that PA plays a central role in intermediary metabolism in the human body. Further esterification of PA can occur in the gut via the reaction with ethyl alcohol generated by the same bacteria (see below). In terms of the breath release, bacteria can't appear in the blood stream because of the gut-blood barrier, but their products like PA or esters of PA – can, similarly to esters of butyric acid. It is known that PA is a weak molecule not stable in aqueous solution, but esters of PA are much more stable, leading thus to the

possibility of their transportation through the bloodstream with further release via exhaled air in the lungs alveoli. We speculate that PA in our case is mostly the product of *Lactobacillis,* which can be attributed to good bacteria present in stomach. Their strains have different susceptibility to antibiotics. For example, several of those in [3] are resistant to metronidazole but susceptible to tetracycline [52], a component of QAC. We excluded *Enterococcus faecium,* also present in the probiotic list [3] because it demonstrates resistance against both just mentioned antibiotics [53,54]. *Bifidobacterium,* another candidate from the probiotics list [51], does not provide the necessary metabolism whereas the metabolism of *Lactobacillis* includes PA and ethanol synthesis leading to the natural production of ethyl pyruvate [55].

<u>3.2. Butyric acid</u> is another short-chain fatty acid, representing an end product of the food fermentation solely performed by anaerobic bacteria in the gut such as *Anaerostipes, Coprococcus, Eubacterium, Faecalibacterium, Clostridium* and *Roseburia* [60,61]. The main scenario for generating esters of butyric acid can be realized directly in the gastrointestinal tract by anaerobic bacteria, with their further efficient release to the blood stream. Bacteria of class *Clostridia* efficiently produce BA [58,59] and methanol, both in frame of the carbohydrate metabolism. Considering the bacteria candidates from those mentioned above, we chose *Eubacterium* that belongs to class *Clostridia* as the main source of the signal at 1170 $cm^{-1}$ and 2972 $cm^{-1}$ (see section 3.2). Two more arguments pro *Eubacterium*: a) it is known that the bacterium is elevated for the *Hp*-infected cases [60] explaining in such a way the point at day "-2" , b) it has high resistivity to tetracycline but reasonably high susceptibility to metronidazole (a part of QAC [61]), representing thus semi-resistant bacteria.

3.3. <u>Propionic acid</u> (1189 $cm^{-1}$) and ethyl vinyl ketone (1203 $cm^{-1}$): we speculate that the corresponding parent bacterium resistant to QAC is *Enterococcus faecium*.

**4. A transportation scheme for methyl butyrate and ethyl pyruvate in the body.**

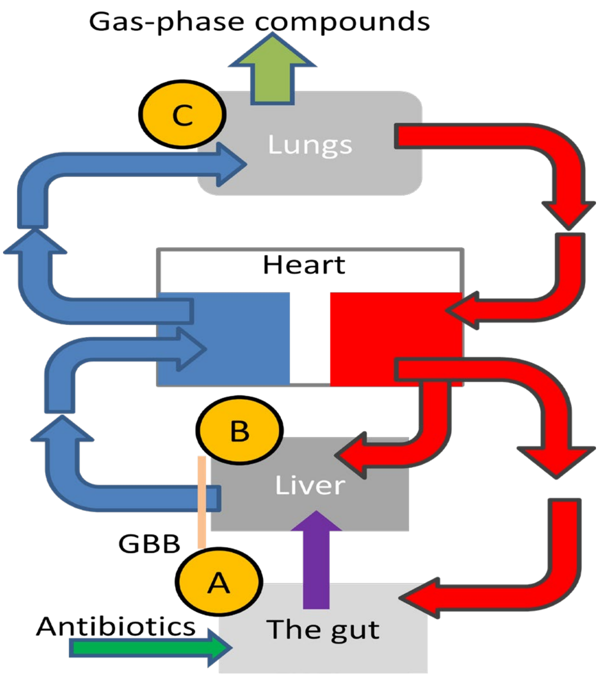


*Figure S2. Working hypothesis of transportation of methyl butyrate and ethyl pyruvate in the body, with their release via exhaled air in lungs. Pathway A-B-C connects the gut where antibiotics come, with the liver and the lungs via veins (shown by blue arrows). Red arrows show arteries, purple arrow shows the bloodstream containing a mixture of arterial and venous blood between the gut and the liver. GBB: the gut-blood barrier semi-transparent to pyruvic and butyric acids and their esters..*
